# Using Employment Data From a Medical University to Examine the Current Occupation Situation of Master's Graduates in Public Health and Preventive Medicine in China

**DOI:** 10.3389/fpubh.2020.508109

**Published:** 2020-12-02

**Authors:** Huangyuan Li, Fuli Zheng, Jie Zhang, Zhenkun Guo, Hua Yang, Caixia Ren, Wenchang Zhang, Siying Wu

**Affiliations:** ^1^Department of Preventive Medicine, School of Public Health, Fujian Medical University, Fuzhou, China; ^2^Office of Student Affairs, School of Health, Fujian Medical University, Fuzhou, China; ^3^The Key Laboratory of Environment and Health, School of Public Health, Fujian Medical University, Fuzhou, China; ^4^Office of Education, School of Public Health, Fujian Medical University, Fuzhou, China; ^5^Department of Epidemiology and Health Statistics, School of Public Health, Fujian Medical University, Fuzhou, China

**Keywords:** public health and preventive medicine, master graduates, employment status, existing problems, suggestions

## Abstract

**Aims:** The purposes of this study are (1) to understand the current employment situation of master's graduates in Public Health and Preventive Medicine (PHPM) and (2) to provide evidence for career guidance and training of competent PHPM personnel.

**Methods:** The master's graduates of the School of Public Health from the years 2014–2018 who majored in PHPM were chosen as research subjects. Questionnaires were distributed, and completed questionnaires were collected. The employment situation and characteristics of these graduates were analyzed based on the questionnaire data.

**Results:** The employment rate of these graduates was 95.45%. They were employed mainly in hospitals, followed by colleges and centers for disease control and prevention. The initial salaries were low. Graduates whose jobs barely or not at all matched their areas of specialization were 23.64 or 6.36%, respectively. Nevertheless, the percentage of students who had remained with their jobs since graduation was 82.73%. A total of 40% of the graduates were neutral about or dissatisfied with their jobs. Furthermore, 29% of them claimed that they were undervalued by their employers. Last, but not least, graduates were encouraged to gain experience in creativity, organizational or management skills, social networking experience, and interpersonal and professional skills.

**Conclusion:** Overall, the employment status of master's graduates in PHPM is good. Problems such as low initial salaries, jobs not matching graduates' areas of specialization, and feelings of being undervalued by employers were observed. It is necessary to improve employment outcomes by revising training models, formulating employment policies, and implementing training efforts.

## Introduction

Public Health and Preventive Medicine (PHPM) is one of the fundamental curricula of modern medicine. As the most important component of medicine, its concept runs through the entire history of humans and diseases, and it has played a major role in the prevention and control of infectious diseases. The field and content of public health have developed considerably. However, new challenges have arisen in health promotion due to the changes in population structure, the ecosystem, and lifestyle (1). In 2002 and 2003, the outbreak and spread of SARS drew great attention to the field of public health and the prevention of infectious disease. It also emphasized the importance of the education and training of PHPM personnel to respond to such emergencies (2). *Healthy China 2030 Planning Outline*, issued by the CPC Central Committee, offers a blueprint for the construction of a healthy China and guidance for the development of the public health field, including the establishment of training programs for high-level personnel.

Public health has become an indispensable component of health education in China, due to the prevention-oriented policy of the Chinese government (3). The demands for both the quantity and the quality of personnel specialized in PHPM to adapt to the developing and future challenges of disease prevention and control have increased. Additional problems, such as aged teams and varying developments among regions, have been observed by the centers for disease control (CDCs) (4, 5). With the expanding scale of medical education and the increasing demand to address the core competencies of public health, the need to cultivate competent PHPM personnel has become more pressing. However, the scale of public health education has merely expanded, compared with the rest of the curricula (6). Accompanied by the rapid expansion of the graduate education scale across the country, the medical school in this study has increased the enrollment scale of PHPM graduates. Therefore, understanding the current situation of the PHPM employment outcome is of considerable importance for graduate education.

Extensive studies conducted on graduate education both domestically and internationally have focused mainly on the current education and cultivating models (7–10). However, few have investigated the employment outcome of the master's graduates. In this study, we collected and analyzed current employment outcome data of master's graduates majoring in PHPM at a medical University from the years 2014 to 2018. From the results gathered, we examined the existing problems and suggested corresponding measures to improve the quality of the personnel. Our study will provide evidence for the need for colleges to cultivate various talents in PHPM students.

## Methods

Graduates in five PHPM disciplines (epidemiology and health statistics, labor and environmental hygiene, nutrition and food hygiene, health toxicology, and maternal, child, and adolescent health) from the years 2014 to 2018 were chosen as research subjects. All graduates were from the same university, the Medical Center in the Western Taiwan Straits Economic Zone.

Based on the literature, we designed the questionnaire of employment outcomes for graduates majoring in PHPM. The questionnaire was further modified after consulting experts and teachers in the field. The questionnaire was then pilot tested by 30 master's graduates who graduated from 2012 to 2013 in the field, and revisions were made to the language.

The questionnaire consisted of 36 questions. There was a brief introduction to research design. Information was requested in three areas: (1) basic personal information; (2) employment information (the type of employer, administrative level of the employer, job duties); and (3) career development (salary levels, the stability of employment, occupational competency, and value of employees by the employers).

The questionnaire was imported to an online questionnaire platform for distribution. The cluster sampling method was used to choose 110 master's graduates in PHPM at a medical University from the years 2014 to 2018. The questionnaire was distributed by the class advisor through social media platforms such as WeChat and QQ, which the graduates continued to use after graduation. At this stage, an initial oral consent to participate in the study was obtained from each participant, using the voice function of QQ or WeChat. A total of 110 subjects returned a completed questionnaire, an overall response rate of 100%. Questionnaires were submitted anonymously and numbered according to the time submitted.

The data collected were used to create a database utilizing the Epi Data 3.0 program. The data were stored in a private off-line database owned by the university. Statistical analysis was performed using the SPSS 24.0 software package. The level of statistical significance was *P* < 0.05.

## Analysis of Research Results

### Basic Information of Research Subjects

A total of 110 questionnaires were distributed and collected, with a recovery rate of 100%. All were valid. As shown in Table 1, 42 questionnaires, or 38.18%, were from male graduates The number of graduates were 13 (11.82%), 25 (22.73%), 24 (21.82%), 24 (21.82%), and 24 (21.82%), in the years 2014 to 2018, respectively. The number of subjects majoring in epidemiology and health statistics was the highest, 66 (60%), while the number majoring in maternal and adolescent health was the lowest, 3 (2.73%). Most of the subjects (95.45%) chose to find a job directly after graduation, while 3.64% decided to pursue postgraduate studies, both domestically and abroad.

**Table 1:** Basic information for PHPM Master's graduates.

**Item**	**Categories**	***N* (persons)**	**Percentage (%)**
Gender	Male	42	38.18
	Female	68	61.82
Graduation year	2014	13	11.82
	2015	25	22.73
	2016	24	21.82
	2017	24	21.82
	2018	24	21.82
Discipline	Epidemiology and health statistics	66	60.00
	Labor and environmental hygiene	15	13.64
	Nutrition and food hygiene	12	10.91
	Health toxicology	14	12.73
	Maternal, child, and adolescent health	3	2.73
Choice after graduation	Direct employment	105	95.45
	Pursue further studies	4	3.64
	Self-employed	0	0.00
	Other	1	0.91

### Current Employment Situation

The type of employer is a key concern of graduates seeking jobs. As shown in Table 2, the master's graduates were mainly employed by hospitals (38.18%), followed by colleges (22.73%), then CDCs (21.82%). When analyzing administrative levels of the employers, provincial (47.92%), and municipal levels (37.5%) employed most of the graduates. More than half of the graduates (64.55%) were engaged in professional and technical work, while the remaining 35.45% occupied other positions.

**Table 2:** Current employment situation of Master's graduates in PHPM.

**Item**	**Categories**	***N* (persons)**	**Percentage (%)**
Type of employer	CDC	24	21.82
	Public health–related institutes other than CDCs	5	4.55
	Hospitals	42	38.18
	Colleges and universities	25	22.73
	Enterprises	7	6.36
	Other	7	6.36
Administrative level of employer	National	2	2.08
	Provincial	46	47.92
	Municipal level	36	37.50
	County (district) level	10	10.42
	Other	2	2.08
Job duty	Administration	27	24.55
	Professional	71	64.55
	Logistical	3	2.73
	Other	9	8.18

### Career Development

Career development is a process that helps shape a person's work identity. Here, we investigated the career development status of the research subjects, such as their salary levels, employment stability, occupational competency, and their perception of how they were valued by employers.

#### Salary Levels

Salary is one of the key indices of the quality of employment. As Table 3 shows, 30.84% of the graduates were paid <4,000 yuan as their initial salaries. Further investigation revealed that there was no significant difference in the initial salary of graduates regardless of discipline and type of work. However, working for different types of employers and for different administrative levels resulted in significant differences in the initial salaries.

**Table 3:** Initial salaries of PHPM Master's graduates.

**Item**	**Categories**	**Initial salaries (yuans)**				**χ^**2**^/*P***
		**Below 3,000**	**3,000–4,000**	**4,000–6,000**	**Over 6,000**	
Discipline	Epidemiology and health statistics	11	15	22	18	χ^2^ = 12.224
		(16.7%)	(22.7%)	(33.3%)	(27.3%)	*P* = 0.428
	Labor and environmental hygiene	2	1	5	6	
		(14.3%)	(7.1%)	(35.7%)	(42.9%)	
	Nutrition and food hygiene	1	1	10	3	
		(6.7%)	(6.7%)	(66.7%)	(20.0%)	
	Health toxicology	1	1	4	6	
		(8.3%)	(8.3%)	(33.3%)	(50.0%)	
	Maternal, child, and adolescent health	0	1	1	1	
		(0%)	(33.3%)	(33.3%)	(33.3%)	
Type of employer	CDC	7	7	8	2	χ^2^ = 32.486
		(29.2%)	(29.2%)	(33.3%)	(8.3%)	*P* = 0.006
	Public health—related institutes other than CDCs	1	2	1	1	
		(20.0%)	(40.0%)	(20.0%)	(20.0%)	
	Hospitals	5	7	14	16	
		(11.9%)	(16.7%)	(33.3%)	(38.1%)	
	Colleges and universities	2	3	15	5	
		(8.0%)	(12.0%)	(60.0%)	(20.0%)	
	Enterprises	0	0	1	6	
		(0%)	(.0%)	(14.3%)	(85.7%)	
	Other	0	0	3	4	
		(0%)	(0%)	(42.9%)	(57.1%)	
Administrative level of employer	National	1	0	0	1	χ^2^ = 26.523
		(50.0%)	(0%)	(0%)	(50.0%)	*P* = 0.009
	Provincial	9	8	21	8	
		(19.6%)	(17.4%)	(45.7%)	(17.4%)	
	Municipal level	3	10	14	9	
		(8.3%)	(27.8%)	(38.9%)	(25.0%)	
	County (district) level	2	1	2	5	
		(20.0%)	(10.0%)	(20.0%)	(50.0%)	
	Other	0	0	5	11	
		(0%)	(0%)	(31.3%)	(68.8%)	
Job duty	Administration	4	2	13	8	χ^2^ = 11.311
		(14.8%)	(7.4%)	(48.1%)	(29.6%)	*P* = 0.255
	Professional	9	15	25	22	
		(12.7%)	(21.1%)	(35.2%)	(31.0%)	
	Logistical	0	2	1	0	
		(0%)	(66.7%)	(33.3%)	(0%)	
	Other	2	0	3	4	
		(22.2%)	(0%)	(33.3%)	(44.4%)	

#### Stability of Employment

Matching area of specialization to job is an important part of the fulfillment of human capital investment. As presented here, 70% of the subjects believed that their jobs matched their areas of specialization, while 30% claimed that their employment barely or not at all matched their specializations. Of the total research subjects, 82.73% did not change their employment after graduation. Those who made job moves declared that they did so because they “didn't have enough space to develop” (42.11%), were “looking for higher salaries” (26.32%), or “would like to change jobs or disciplines” (26.32%). Overall, 40% of the graduates were neutral about or dissatisfied with their jobs (Table 4).

**Table 4:** Employment stability of PHPM Master's graduates.

**Item**	**Categories**	***N* (persons)**	**Percentage (%)**
Extent of jobs matching specializations	Totally	33	30.00
	Mainly	44	40.00
	Barely	26	23.64
	Not at all	7	6.36
Number of job switches	Never	91	82.73
	Once	17	15.45
	Twice	2	1.82
	Three times or more	0	0.00
Reason for changing jobs	Limited opportunity for personal development	8	42.10
	Pursuit of higher salaries	5	26.32
	Seeking job or discipline changes	5	26.32
	Other	1	5.26
Satisfactory with employment	Very satisfied	16	14.55
	Satisfied	50	45.45
	Neutral	39	35.45
	Not satisfied	4	3.64
	Extremely disappointed	1	0.91

#### Occupational Competency and Value by Employers

As shown in Table 5, 44.55 and 45.45% of the research subjects suggested they were either totally or mainly capable of performing their jobs. As to the abilities and qualities that help them quickly adapt to their jobs, the highest-ranked ability was “willing to go through hardships” (76.36%), followed by “outstanding professional skills” (65.45%), then “wide range of knowledge” (43.64%). However, 29, 2, and 1 person(s) claimed that they were merely, not, or considerably not valued by their employers, respectively. Regarding the qualities or abilities they believed they lacked, nearly half of them named creativity (48.18%), followed by organizational skills and management (43.73%), social networking skills (35.45%), interpersonal skills (30.00%), and professional skills (25.45%). A total of 25 subjects believed they lacked psychological sustainability.

**Table 5:** Occupational competency and value by employers of PHPM Master's graduates.

**Item**	**Categories**	***N* (persons)**	**Percentage (%)**
Occupational competency	Totally capable	49	44.55
	Mainly capable	50	45.45
	Neutral	11	10.00
	Not capable	0	0.00
Adaption to jobs based on	Outstanding professional skills	72	65.45
	Willing to go through hardships	84	76.36
	Innovation and creativity	25	22.73
	Extensive interpersonal skills	30	27.27
	Wide range of knowledge	48	43.64
	Other	21	19.09
Value by employers	Extremely	10	9.09
	Relatively	68	61.82
	Not much	29	26.36
	Barely	2	1.82
	Not valued	1	0.91
Ability or skill that is lacking mostly	Professional or practical skills	28	25.45
	Social networking skills	39	35.45
	Creativity	53	48.18
	Interpersonal skills	33	30.00
	Team-working ability	13	11.82
	Organizing and management ability	47	42.73
	Psychological sustainability	25	22.73
	Other	12	10.91

## Discussion

### The Employment Rate of Master's Graduates Remains at a Relatively High Level, but Many Are Working in Areas Unrelated to Their Majors

Most chose to start work immediately, and only a small number decided to pursue further studies. The employment area is mainly in the province where the University is located and concentrated in coastal cities. This may be due to the working environment, relatively high income level, and more job opportunities for highly educated employers. Due to the need to cultivate professional personnel in the PHPM field, the graduates belong to the specialist team (11). A majority of public health graduates are employed by CDCs, health inspection institutes, or hospitals in China (12, 13). However, in this study, the number working in CDCs ranked third, after colleges, and significantly lower than the numbers employed in hospitals (where the most graduates were employed). Nearly 35% of the graduates were doing administrative or logistical work unrelated to their specializations. Our results imply that this preference of hospitals and universities over CDCs leads to the loss of high-caliber personnel in the PHPM field. Interestingly, in the study of the occupational situation of master's graduates in PHPM in Fudan University and Xuzhou Medical University (representing key University and average university) showed that 40–50% of the master's graduates were employed by hospitals, followed by CDCs and colleges, which was similar to our results (14, 15). According to J.P. Leider (16), since the vast majority of people are not engaged in government public health work, state and local health departments continue to report severe labor shortages, although the number of graduates in public health has increased. Moreover, a large number of the graduates were employed by provincial- or municipal-level institutions, which resulted in the relatively low education level in rudimentary CDCs.

### Lower Salary May Distract Graduates From Working in CDC

Salary is one of the most important factors for job seekers. Along with medical reform, the income of medical staff in hospitals has increased. Nevertheless, as is the nature of a public institution, the CDCs pay relatively low salaries based on standard and performance. Graduates could earn more in hospitals, colleges, and enterprises than in CDCs or other public health–related institutes, which could be one of the reasons that prevent them from working in disease prevention–related institutes. The initial salary of the master's graduates from various administrative levels varied. Only 30.91% of the graduates have a monthly income of over 6,000 yuan. Working in provincial or municipal cities with higher living expenses, the graduates often found their initial salaries merely met their expectations. Therefore, nearly 20% of respondents have changed jobs, and 26.32% of them are in pursuit of higher salaries. Owing to this, the satisfaction rate of staff in the CDCs and their passion for work were inevitably affected. Salaries are an important part of the recruitment and retention of public health personnel (15). Financial incentives may be important determinants of worker motivation and intention to leave (17–21). These results are consistent with previous studies of job satisfaction in the field of Public Health in settings elsewhere (22–25). Improving pay and benefits has become an urgent issue to be resolved in recruiting PHPM personnel.

### It Is Worth Paying Attention to the Current Situation of Professional Mismatch and Job Dissatisfaction

Whether jobs fit graduate specializations is a superficial demonstration in terms of employment quality. Since PHPM is a field of strong professionalism, during the job-seeking processes, a majority of graduates would initially target the jobs in their areas of specialization. As a consequence, the jobs they found were generally in the PHPM field. Nevertheless, 30.00% of the graduates still claimed their jobs did not match their areas of specialization. It is believed that job satisfaction for health workers is important since it is related to internal motivation and overall job performance. Although 82.73% of health workers have not changed jobs, 40.00% of the subjects stated that they were not satisfied or barely satisfied with their current employment, suggesting that there is a gap between their expectations and current situations. Job dissatisfaction is caused by multiple factors, including salary and incentives, management and communication within the organization, and opportunities for training and promotion (22, 26). It is worth pointing out that the ideal major for many was clinical medicine, but due to the limited score and passive adjustment, they ended up in PHPM. These students were more likely to find a job irrelevant to PHPM as they were not satisfied with their major in the first place. Indeed, one study showed that ~1–5th of graduates were unwilling to choose a career in the PHPM field in China (27).

### Master's Graduates Are Qualified for the Job, but Some of Them Are Not Valued

As the findings of employment adaptation demonstrated, the graduates were mostly competent for their jobs. In addition, the determination to endure hardship, professional and practical skills, and a wide range of knowledge from University training contributed to solid foundations in employment. But improvements in creativity, organizational and management abilities, mental sustainability, social networking, and interpersonal skills are needed. Similarly, studies in the United States and elsewhere strengthened the importance of embedding skill training in professional training, including leadership, project management, communication, problem-solving, planning, finance, and process improvement (28–31). Moreover, public health managers aiming to improve levels of job satisfaction should focus on workforce development and training efforts as well as adequate supervisory support, especially for new hires (24). As shown here, nearly 29% of the research subjects felt that they were merely valued by their employers. This sense of feeling undervalued, in turn, could lead to the dissatisfaction found by the questionnaire.

Based on the analysis above, we promoted the following political suggestions to improve the quality of employment of master's graduates in public health and preventive medicine. In formulating policies on talent recruitment, the appeal of salaries and benefits should be taken into account. Reasonable determinations of the compensation that reflects the value and professional contribution of public health personnel are required. Moreover, provincial governments should fully understand the importance of PHPM talent recruitment and cultivation, and provide adequate conditions for the introduction and preservation of personnel. This includes strengthening in various areas such as working environment, salary levels, personnel file management, training systems, continuing education, and promotion channels. For colleges and universities, a better understanding of the requirements of PHPM personnel from the developing society is of considerable importance. Colleges should formulate various training aims and methods to optimize the scale of education and cultivation system (32, 33). To increase sustainable development, the construction of curricula, the management of cultivation, and skill training should be stressed. Not only the research and innovation abilities should be cultivated, but also non-academic abilities such as teamwork, communication, and leadership are needed, so that the graduates can adapt to the diversified needs of the employment market (16, 20–24, 34). Furthermore, conducting a follow-up survey on the employment and career development of the master's in public health helps accurately grasp the development and utilization of graduates' employment orientation and employability, which in turn serves as feedback and guidance for the training of PHPM master's and employment services. Last, but not least, the employment departments and instructors of universities and public health institutions should (1) assist graduate students in career planning and job search guidance; (2) give full play to the role of academic associations, academic groups, and industry associations; and (3) build a master's employment information platform to promote the psychological and role transformation from schools to occupations.

## Conclusion

In conclusion, the employment outcomes of public health master's graduates are in a good situation, with relatively stable employment and job satisfaction. However, problems have been observed, such as generally low initial salaries, poor fit of employments, and undervaluing of employees by the employers. In the context of expanding the scale of master's students and developing the career of public health, the promotion of high-quality employment of these graduates is of significant importance. To avoid waste of education resources and talent, we should make efforts to improve the cultivation model, to the benefit of both employers and employees.

## Data Availability Statement

The datasets generated for this study are available on request to the corresponding author.

## Ethics Statement

Ethical review and approval was not required for the study on human participants in accordance with the local legislation and institutional requirements. The patients/participants provided their written informed consent to participate in this study.

## Author Contributions

HL, FZ, JZ, ZG, HY, CR, WZ, and SW made intellectual contributions to this study. HL, FZ, and JZ designed and wrote most of the paper and conducted the data analysis with assistance from SW. ZG, HY, CR, and WZ were major contributors to data collection and paper revision. All authors reviewed and approved the draft of the manuscript and ensured the accuracy and integrity of the work before submission.

## Conflict of Interest

The authors declare that the research was conducted in the absence of any commercial or financial relationships that could be construed as a potential conflict of interest.
